# Non-linear scaling of a musculoskeletal model of the lower limb using statistical shape models

**DOI:** 10.1016/j.jbiomech.2016.09.005

**Published:** 2016-10-03

**Authors:** Daniel Nolte, Chui Kit Tsang, Kai Yu Zhang, Ziyun Ding, Angela E. Kedgley, Anthony M.J. Bull

**Affiliations:** Department of Bioengineering, Imperial College London, London SW7 2AZ, United Kingdom

**Keywords:** Lower extremity, Musculoskeletal model, Scaling methods, Subject-specific modelling, Statistical shape modelling

## Abstract

Accurate muscle geometry for musculoskeletal models is important to enable accurate subject-specific simulations. Commonly, linear scaling is used to obtain individualised muscle geometry. More advanced methods include non-linear scaling using segmented bone surfaces and manual or semi-automatic digitisation of muscle paths from medical images. In this study, a new scaling method combining non-linear scaling with reconstructions of bone surfaces using statistical shape modelling is presented. Statistical Shape Models (SSMs) of femur and tibia/fibula were used to reconstruct bone surfaces of nine subjects. Reference models were created by morphing manually digitised muscle paths to mean shapes of the SSMs using non-linear transformations and inter-subject variability was calculated. Subject-specific models of muscle attachment and via points were created from three reference models. The accuracy was evaluated by calculating the differences between the scaled and manually digitised models. The points defining the muscle paths showed large inter-subject variability at the thigh and shank *–* up to 26 mm; this was found to limit the accuracy of all studied scaling methods. Errors for the subject-specific muscle point reconstructions of the thigh could be decreased by 9% to 20% by using the non-linear scaling compared to a typical linear scaling method. We conclude that the proposed non-linear scaling method is more accurate than linear scaling methods. Thus, when combined with the ability to reconstruct bone surfaces from incomplete or scattered geometry data using statistical shape models our proposed method is an alternative to linear scaling methods.

## Nomenclature

FFD: Free Form Deformation
FM: Medium female subject
FS: Small female subject
FT: Tall female subject
IRTK: Image Registration Toolkit
ISB: International Society of Biomechanics
MM, MM2: Medium male subjects
MR: Magnetic Resonance
Mref: Male reference subject
MS: Small male subject
MT, MT2: Tall male subjects
OI: Origin and insertion
PMV: Principal mode of variation
RSME: Root Mean Square Error
SD: Standard Deviation
SSM: Statistical Shape Model
SSMRT: Statistical Shape Modelling Research Toolkit

## 1 Introduction

Knowledge of the distribution and magnitude of forces in the musculoskeletal system relies on accurate quantification of muscle forces. This knowledge can be used to investigate all mechanically-mediated conditions and interventions in the musculoskeletal system, including, osteoarthritis, implants, fracture fixation devices, rehabilitation and athletic performance. Several methods to create subject-specific models of muscle geometries of the lower limb have been published. The methods include linear scaling (Cleather and Bull, 2010, Correa and Pandy, 2011, Lund et al., 2015, Sommer et al., 1982), non-linear scaling based on bone geometries (Kaptein and van der Helm, 2004, Pellikaan et al., 2014) and semi-automatic (Scheys et al., 2005) and manual digitisation (Correa et al., 2011, Ding et al., 2015) of medical images. Kaptein and van der Helm (2004) found that more than 50% of the scapula muscle paths could be reconstructed with high accuracy using a non-linear scaling method. Pellikaan et al. (2014) found that a non-linear morphing algorithm based on digitised bone geometries was able to morph muscle attachment sites between digitised scans of two cadavers with average errors smaller than 15 mm for almost 70% of the muscle attachment points. A common limitation of non-linear scaling methods is the need for either segmented bone surfaces or medical images of the entire limb. This limits the applicability of these methods for musculoskeletal analysis when image data are not available.

Statistical shape models (SSMs) allow accurate reconstruction of geometries from sparse data obtained with basic clinical imaging techniques. These include reconstruction of a 3D shape from a single X-ray (Zheng and Nolte, 2006) or stereo X-ray (Baka et al., 2011) as well as the prediction of a healthy from a pathological shape from 3D scans of joint regions (Rajamani et al., 2004, Rajamani et al., 2005). Linking together bone morphing using reconstructions and geometrical models of muscle paths has not been attempted previously.

In this study, the accuracy of a non-linear scaling method using bone morphing between shapes of nine different subjects reconstructed using a statistical shape modelling toolkit was investigated. Results of muscle paths and landmarks were compared to linearly scaled models using two methods: a landmark-based scaling method, to represent approaches used in the literature, and an affine scaling method minimising the distance between two bone surfaces, to estimate the lower bound for errors that are obtained from an arbitrary linear scaling law. The hypothesis of the study was that non-linearly scaled models created using statistical shape modelling significantly decrease the error between reconstruction and manual digitisation compared to linearly scaled models.

## 2 Material and methods

### 2.1 Subjects

The study was approved by the Imperial College Research Ethics Committee and all subjects provided written informed consent. Magnetic Resonance (MR) imaging scans using a 3.0 T MR scanner (MAGNETROM Verio, Siemens, Germany) with a slice thickness of 1 mm and an in-plane resolution of 1.406 mm×1.406 mm were obtained of 35 subjects. Additionally, lower limb Computed Tomography scans of eight subjects were used for bone surface segmentations. Bone surfaces of the femur and tibia/fibula were segmented of all subjects using a semi-automatic procedure. For nine of the MR scanned subjects (Table 1) paths of 38 muscles and the patellar ligament were digitised with 163 polygonal line elements in total with origin/insertion and via points following the topology described in Klein Horsman et al. (2007). Further, tibiofemoral contact points, joint centres of rotation and bony landmarks used to create local reference frames of the segments (Table 2), following the ISB recommendations (Wu et al., 2002), were digitised. The digitisations and segmentations were performed using Mimics (Mimics 17.0, Materialise, Belgium) by one imaging expert.

**Table 1:** Detailed information of nine subjects used for manual digitisations of muscle geometries. Subject labels describe the gender (M/F) and an attribute (S: small, M: medium, T: tall, ref: reference).

**Subject**	**Gender**	**Height (cm)**	**Mass (kg)**	**Femur length (mm)**	**Tibia/Fibula length (mm)**	**Pelvis width (mm)**	**Age (years)**
**MT2**	Male	183	96	428.3	441.6	227.9	42
**MS**	Male	168	64	377.1	384.6	229.4	21
**FM**	Female	168	70	418.2	414.6	220.8	45
**FS**	Female	155	45	345.9	366.2	230.8	27
**MT**	Male	192	85	460.9	465.8	245.0	27
**Mref**	Male	172	70	407.4	410.4	235.4	35
**FT**	Female	184	78	446.5	455.4	246.9	43
**MM**	Male	180	70	418.4	425.5	218.6	25
**MM2**	Male	175	76	443.7	450.7	219.5	25

**Table 2:** List of landmarks digitised on the bone geometry with descriptions of their location.

**Pelvis**	**RASIS/LASIS**	Right/left anterior superior iliac spine
	**RPSIS/LPSIS**	Right/left posterior superior iliac spine
**Thigh**	**RLFE/LLFE**	Right/left lateral femoral epicondyle
	**RMFE/LMFE**	Right/left medial femoral epicondyle
**Shank**	**RMM/LMM**	Right/left medial malleolus
	**RLM/LLM**	Right/left lateral malleolus
**Foot**	**RFCC/LFCC**	Right/left calcaneus (heel)
	**RMF2/LFM2**	Right/left head of second metatarsal
	**RFMT/LFMT**	Right/left tuberosity of fifth metatarsal

### 2.2 SSMs and bone surface reconstructions

SSMs of femur and tibia/fibula were constructed from 68 bone geometries of the right and mirrored left leg of the 34 subjects not used to digitise muscle geometries. The SSMs were created using a construction pipeline which aligns and registers surfaces using rigid-body transformations and calculates modes of variation using principle component analysis (described in Zhang et al., 2012). The morphological variation of femur and tibia were well represented, as illustrated by the high power of the models: 95% of the population was represented with four and eight principal modes of variation (PMVs) for femur and tibia/fibula, respectively; 98.5% was represented by 16 and 27 PMVs.

For each of the other nine subjects, the femur and tibia/fibula bones were reconstructed from random point sets containing 1000 points, which is less than 10% of the number of points representing the SSM mean shapes. Further, subsets containing only points from the proximal and distal 20% of the bones for a comparison to reconstructions from incomplete medical images were created. For registration, sets of corresponding landmarks were digitised on mean shapes and subject bones. The random points were registered to the mean shape of the SSM using a sequence of landmark-based and surface-based rigid body transformations using the Image Registration Toolkit (IRTK) (Rueckert et al., 1999, Schnabel et al., 2001) with manual corrections when necessary to reduce errors (see Supplementary Material). For the reconstruction, a morphing algorithm adding weighted PMVs to the mean shape of an SSM to minimise the Mahalanobis distance to a point cloud was used (Rajamani et al., 2005, Yang et al., 2008). Non-linear B-spline Free-Form Deformations (FFDs) with a node spacing of 20 mm using the IRTK for the mappings between mean shapes and reconstructed surfaces were calculated. All algorithms for creating and morphing SSMs together with the SSM are available as Statistical Shape Modelling Research Toolkit (SSMRT) at http://www.msksoftware.org.uk. The reconstruction quality of the bones was evaluated by calculating the RMSE between the manually segmented and the reconstructed bone surfaces using Geomagic Studio 12 (Geomagic, Inc., USA).

### 2.3 Reference and subject-specific muscle models

To create reference muscle paths from all nine subjects, FFDs from subject to mean shape were applied to the muscle paths and landmarks. For comparison, muscle paths, bone surfaces and landmarks were scaled to the mean surfaces of the SSM using a two-parameter linear and an affine scaling method. The linear scaling method used segment lengths and pelvis width as scaling factors (Table 3). The affine scaling method minimised the least-squares distance between two surfaces using an affine transformation. Since this method used information of the complete bone surface, it is considered as a lower bound for the error of linear scaling methods using bony landmarks or other bone dimensions. The accuracy of the transformations was evaluated by calculating the root mean square error (RMSE) between mean shapes and the transformed subject. The variances of muscle geometries were calculated using the FFD transformed geometries of all subjects transformed to the mean shapes.

**Table 3:** Definition of parameters of the linear scaling law: The pelvis width was calculated as the distance between right and left anterior iliac spine landmark; the segment lengths were defined as distance between hip joint centre and the middle between lateral and medial femoral epicondyle landmarks, femoral epicondyle midpoint and midpoint between tibial and fibula malleoli, and mid malleoli and distal end of the second metatarsal.

	**Length**	**Width**
**Pelvis**	Thigh length	Pelvis width
**Thigh**	Thigh length	Pelvis width
**Shank**	Shank length	Pelvis width
**Foot**	Foot length	Pelvis width
**Patella**	Pelvis width	Thigh length

**Fig. 1:**
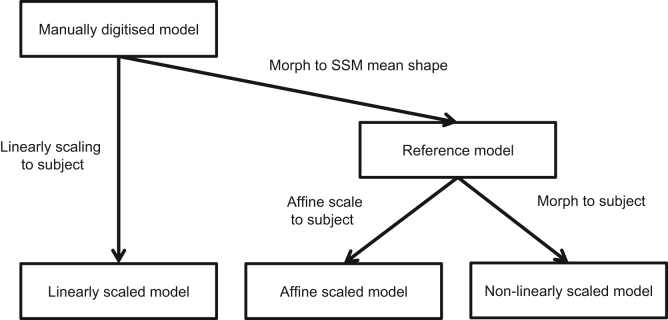
Overview of compared scaling methods to create subject-specific geometrical muscle models. Reference models were created by morphing manually digitised models to mean shapes of SSMs. Subject-specific models were created by linearly scaling from manually digitisations, affine and non-linear scaling from reference models.

Subject-specific landmarks and muscle paths, origin and insertion points were reconstructed from reference models of three different subjects chosen to represent the breadth of morphological differences of the population: a male subject closest to the mean size and weight of all subjects (Mref), the smallest female (FS) and the tallest male subject (MT). All bone models used to create the SSMs were from subjects who fell within the height range of these models. Non-linear and affine transformations from mean shape to subject surface were applied to the reference shapes to reconstruct the geometries (Fig. 1). The linear scaling method was applied to the digitised subject model that corresponded to the reference model employed. The accuracy of the scaling methods was evaluated by measuring the RMSE between manually digitised and scaled models.

### 2.4 Statistical Analysis

Standard deviations were calculated for corresponding muscle points. Analysis of variance was performed (R 3.2.1, www.r-project.org) to evaluate differences between the scaling methods for reference models scaled to the source model as well as for the three representative reference models to the other subjects. We calculated differences between manually digitised and scaled muscle points (including and excluding via points), landmarks and surfaces. Where significance was found paired t-tests using Holm corrections (Holm, 1979) were conducted.

## 3 Results

The bone surfaces were reconstructed with a maximal average error of 1.6±2.0 mm for femur and tibia/fibula bone using the first 50 PMVs. Reconstructions using only points from the distal and proximal bones had a maximal average error of 2.6±3.2 mm using 22 PMVs for the femur and 20 PMVs for the tibia/fibula. Transformations of reconstructed subject-specific to mean surfaces showed significant (*p*<0.05) differences between linear, affine and non-linear scaling methods (Table 4).

**Table 4:** Average RMSE and standard deviation in mm between the mean shape of the SSM and subject surfaces scaled to the mean shape.

	**Linear scaling**	**Affine scaling**	**Non-linear scaling**
	**(L)**	**(A)**	**(N)**
**Thigh**	4.64 (1.66)	3.01 (0.77)	1.29 (0.33)
*Difference*	***L*****>*****A*****,*****p*****=*****8.5e−3***	***A*****>*****N, p=1.1e*****−*****5***	
	***L>N, p=3.4e−4***		
**Shank**	6.82 (1.82)	3.49 (0.67)	1.70 (0.29)
*Difference*	***L>A, p*****=1.5*****e−4***	***A*****>*****N, p*****=*****1.0e*****−*****5***	
	***L>N, p*****=4.8*****e−5***		

Significant differences in bold.

**Fig. 2:**
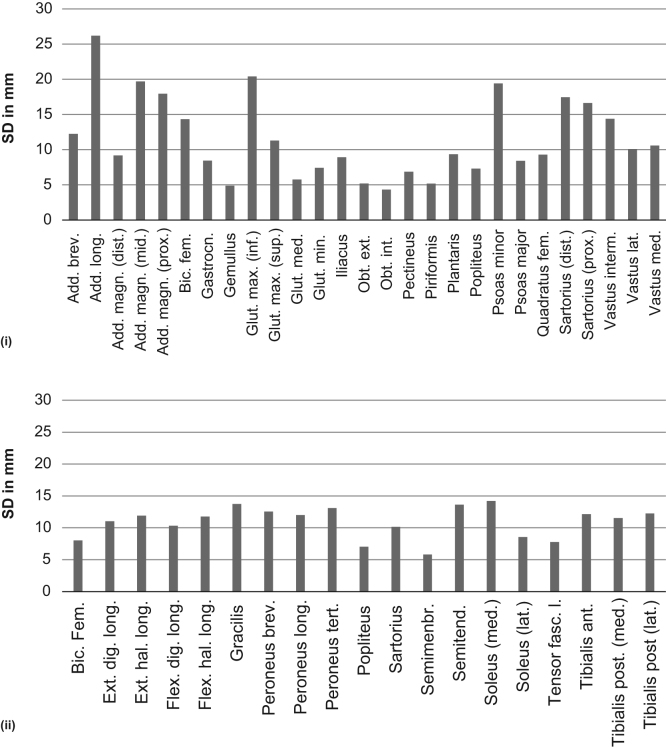
Standard deviations of manually digitised muscle geometries scaled to the mean shapes of statistical shape models of: (i) femur and (ii) tibia/fibula.

**Fig. 3:**
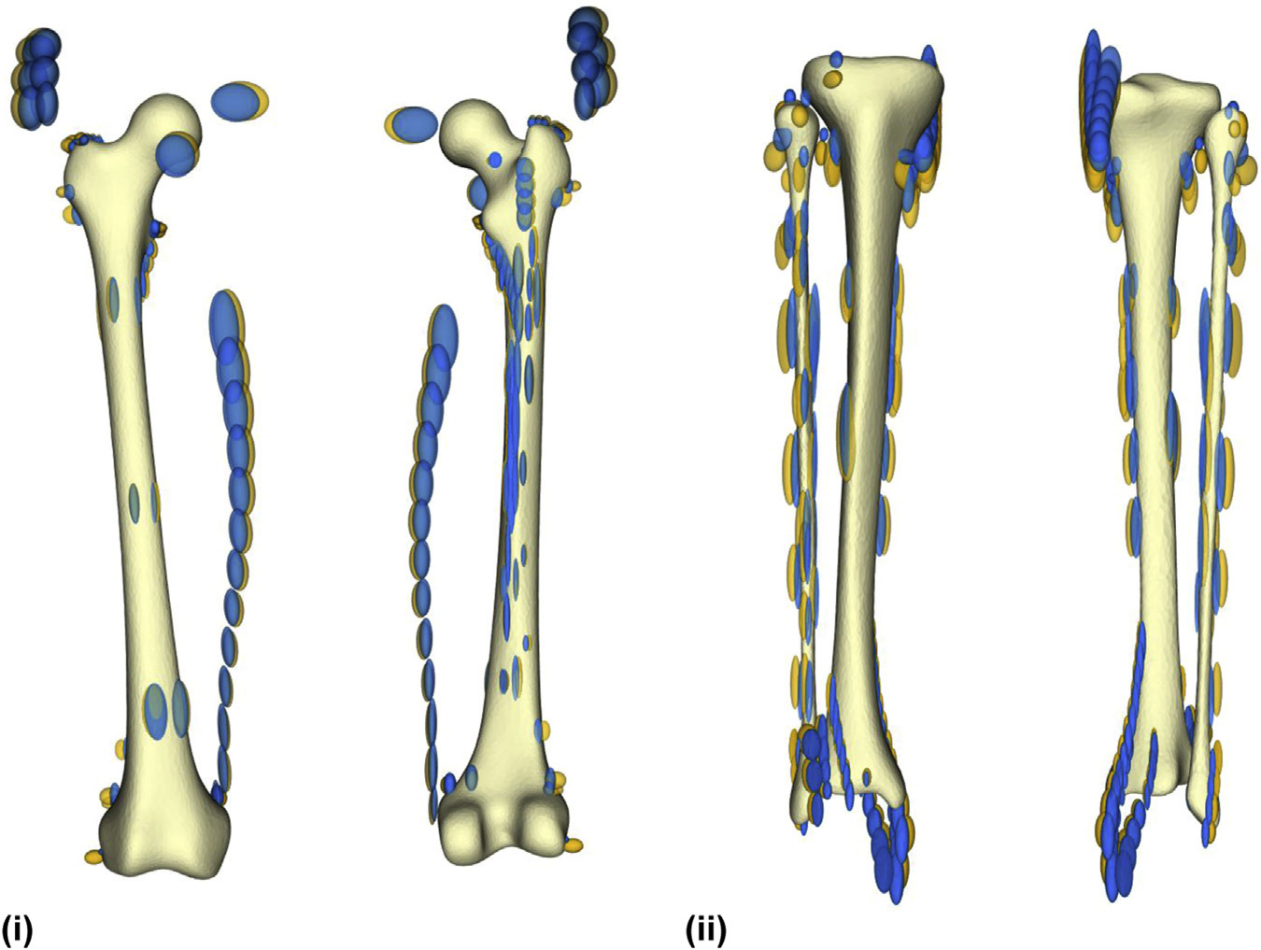
Variance of muscle attachment and via points scaled to the mean shape of (i) thigh and (ii) the shank for linear (yellow) and non-linear scaling (blue). Variances in all three coordinate axes are represented as ellipsoids with axes length scaled with the standard deviation. (For interpretation of the references to color in this figure legend, the reader is referred to the web version of this article.)

The variances between reference muscle geometries are plotted in Fig. 2. The largest variances were observed in the longitudinal direction (Fig. 3). Muscle points and landmarks of a reference morphed to the underlying subject geometry using the three different scaling methods showed similar significant differences between the methods (Table 5; *p*<0.05 for all). Scaling the three reference bone surfaces to each of the other subjects showed significantly lower RMSEs for the non-linear transformation compared to the affine and the linear scaling (*p*<0.05). For muscle geometries, the only significant differences between non-linear scaling and linear scaling were found for the thigh segment (Table 6, *p*<0.05). Average RMSEs of the landmarks did not show any significant differences.

**Table 5:** Average RMSE and standard deviation in mm for the bone surface, muscle paths, muscle origin and insertion (OI) points and landmarks morphed from a reference model to the original subject using two-parameter linear scaling, affine scaling and non-linear scaling.

		**Thigh**	*Difference*		**Shank**	*Difference*	
**Surface**	Linear scaling (L)	4.94 (1.29)	***L*****>*****A, p*****=*****2.1e*****−4**	***L*****>N*****, p*****=4.2*****e*****−6**	6.26 (1.79)	***L>A, p*****=4.8*****e−5***	***L*****>*****N, p*****=1.8*****e*****−*****5***
	Affine scaling (A)	3.23 (1.15)	***A*****>*****N, p*****=1.8*****e−5***		3.60 (1.10)	***A*****>*****N, p*****=2.2*****e−*****5**	
	Non-linear scaling (N)	0.50 (0.33)			0.51 (0.20)		
**Muscle Paths**	Linear scaling (L)	5.29 (1.49)	***L*****>*****A, p*****=1.1*****e−4***	***L*****>*****N, p*****=8.4*****e−*****6**	5.99 (1.54)	***L>A, p=*****2.8*****e−3***	***L>N, p=*****1.0*****e−5***
	Affine scaling (A)	2.67 (1.15)	***A*****>*****N, p*****=5.3*****e−*****5**		4.20 (1.73)	***A>N, p=*****2.6*****e−4***	
	Non-linear scaling (N)	0.64 (0.47)			0.87 (0.40)		
**Muscle OI**	Linear scaling (L)	5.37 (1.46)	***L>A, p=*****2.2*****e−4***	***L*****>*****N, p=8.4e−6***	6.13 (1.64)	***L>A, p=9.8e*****−*****5***	***L>N, p=*****9.5*****e−6***
	Affine scaling (A)	2.82 (1.16	***A*****>*****N, p*****=5.9*****e−5***		3.01 (0.72)	***A>N, p*****=5.2*****e−6***	
	Non-linear scaling (N)	0.54 (0.38)			0.44 (0.16)		
**Landmarks**	Linear scaling (L)	4.95 (1.75)	*L*>*A, p=6.9e**−**2*	***L*****>*****N, p=*****5.4*****e−5***	4.88 (1.20)	*L>A, p=1.8e**−**1*	***L>N, p=*****7.2*****e−6***
	Affine scaling (A)	3.91 (2.18)	***A*****>*****N, p*****=1.0*****e−3***		4.12 (1.64)	***A>N, p*****=1.3*****e−4***	
	Non-linear scaling (N)	0.53 (0.39)			0.83 (0.41)		

Significant differences in bold.

**Table 6:** Average RMSE and standard deviation in mm for the reconstructed surfaces, muscle paths, muscle origin and insertion (OI) points and landmarks for reference models based on subjects Mref, MT and FS.

			**Thigh**	Difference		**Shank**	Difference	
**Mref**	**Surface**	Linear scaling (L)	8.94 (2.96)	***L*>*A*, *p*=6.1e−4**	***L*>*N*, *p*=4.0e−4**	10.08 (2.46)	***L*>*A*, *p*=6.6e−5**	***L*>*N*, *p*=6.6e−5**
		Affine scaling (A)	3.66 (1.09)	***A*>*N*, *p*=5.0e−4**		3.96 (0.78)	***A*>*N*, *p*=6.6e−5**	
		Non-linear scaling (N)	1.96 (0.48)			2.08 (0.31)		
							
	**Muscle Paths**	Linear scaling (L)	17.09 (2.98)	***L*>*A*, *p*=4.5e−2**	**L>N, =4.5e−2**	15.92 (4.25)	*L*>*A*, *p*=1.0	*L*>*N*, *p*=1.0
		Affine scaling (A)	14.41 (1.73)	*A*>*N*, *p*=4.0e**−**1		15.42 (5.26)	*A*>*N*, *p*=1.0	
		Non-linear scaling (N)	14.48 (1.70)			15.18 (4.60)		
							
	**Muscle OI**	Linear scaling (L)	16.76 (2.91)	***L*>*A*, *p*=2.9e−2**	***L*>*N*, *p*=2.9e−2**	20.35 (2.39)	***L*>*A*, *p*=3.8e−3**	***L*>*N*, *p*=4.8e−3**
		Affine scaling (A)	13.69 (1.52)	*A*<*N*, *p*=4.5e**−**1		17.46 (1.54)	*A*<*N*, *p*=6.3e**−**1	
		Non-linear scaling (N)	13.76 (1.55)			17.52 (1.74)		
							
	**Landmarks**	Linear scaling (L)	6.52 (1.71)	*L*<*A*, *p*=9.1e**−**2	*L*>*N*, *p*=1.9e**−**1	9.81 (4.23)	***L*<*A*, *p*=2.1e−2**	*L*<*N*, *p*=8.8e**−**1
		Affine scaling (A)	7.59 (2.51)	*A*>*N*, p=6.0e**−**2		11.16 (4.67)	***A*>*N*, *p*=2.1e−2**	
		Non-linear scaling (N)	6.12 (1.31)			9.88 (4.28)		
								
**FS**	**Surface**	Linear scaling (L)	11.56 (2.32)	***L*>*A*, *p*=1.9*e*−5**	***L*>*N*, *p*=8.7*e*−6**	14.47 (3.31)	***L*>*A*, *p*=3.6*e*−5**	***L*>*N*, *p*=3.6e−5**
		Affine scaling (A)	3.87 (1.14)	***A*>*N*, *p*=2.1*e*−4**		4.76 (0.94)	***A*>*N*, *p*=4.6e−5**	
		Non-linear scaling (N)	2.42 (0.62)			3.46 (0.60)		
							
	**Muscle Paths**	Linear scaling (L)	18.75 (1.66)	**L>A, p=3.7e−3**	**L>N, p=4.0e−3**	19.71 (3.31)	L>A, p=3.2e**−**1	L>N, *p*=2.3e**−**1
		Affine scaling (A)	17.05 (1.36)	A>N, p=2.8e**−**1		17.13 (3.32)	A>N, *p*=3.2e**−**1	
		Non-linear scaling (N)	16.92 (1.26)			16.36 (2.09)		
							
	**Muscle OI**	Linear scaling (L)	18.10 (1.53)	***L*>*A*, *p*=1.8*e*−3**	***L*>*N, p* =2.3*e*−3**	22.65 (1.53)	**L>A, p=8.2e−5**	**L>N, p=1.1e−4**
		Affine scaling (A)	15.98 (1.02)	*A*>*N*, *p*=1.9*e***−**1		17.44 (1.91)	A>N, *p*=3.1e**−**1	
		Non-linear scaling (N)	15.80 (0.89)			17.27 (2.09)		
							
	**Landmarks**	Linear scaling (L)	9.68 (1.45)	***L*<*A*, *p*=3.3e−2**	***L*<*N*, *p*=3.3*e*−2**	12.08 (4.26)	***L*<*A*, *p*=4.6e−2**	*L*<*N*, *p*=1.8*e***−**1
		Affine scaling (A)	12.92 (3.69)	***A*>*N*, *p*=3.3*e*−2**		14.42 (4.50)	***A*>*N*, *p*=4.0e−3**	
		Non-linear scaling (N)	11.62 (2.82)			13.04 (4.16)		
								
**MT**	**Surface**	Linear scaling (L)	10.56 (2.53)	***L*>*A*, *p*=2.7*e*−4**	***L*>*N*, *p*=9.3*e*−5**	9.27 (2.93)	***L*>*A*, *p*=2.7*e*−3**	***L*>N, *p*=2.7*e*−4**
		Affine scaling (A)	3.74 (0.51)	***A*>N, *p*=2.7*e*−4**		4.04 (0.75)	***A*>*N*, *p*=2.6*e*−3**	
		Non-linear scaling (N)	2.39 (0.25)			2.17 (0.46)		
							
	**Muscle Paths**	Linear scaling (L)	20.34 (2.45)	***L*>*A*, *p*=1.6e−2**	***L*>*N*, *p*=1.6*e*−2**	13.91 (2.12)	L>A, p=8.3e**−**1	L>N, *p*=6.5e**−**1
		Affine scaling (A)	16.72 (2.28)	*A*>*N*, *p*=6.4e−2		13.38 (3.10)	A>N, p=8.3e**−**1	
		Non-linear scaling (N)	16.51 (2.09)			12.91 (1.91)		
							
	**Muscle OI**	Linear scaling (L)	19.90 (2.54)	***L*>*A*, *p*=2.2*e*−2**	***L*>*N*, *p*=2.0*e*−2**	16.98 (2.80)	*L*>*A*, *p*=3.3e−1	*L*>*N*, *p*=3.3e−1
		Affine scaling (A)	16.26 (2.68)	*A*>*N*, *p*=6.6e**−**2		15.58 (2.43)	*A*>*N*, *p*=3.9e**−**1	
		Non-linear scaling (N)	16.02 (2.45)			15.39 (2.58)		
							
	**Landmarks**	Linear scaling (L)	7.55 (1.47)	*L*<*A*, *p*=2.7*e*−1	*L*>*N*, *p*=2.7e−1	14.81 (3.39)	*L*<*A*, *p*=1.0	*L*>*N*, *p*=1.0
		Affine scaling (A)	8.00 (2.07)	***A*>*N*, *p*=2.8e−2**		14.91 (3.11)	*A*>*N*, *p*=0.6	
		Non-linear scaling (N)	6.80 (1.66)			14.51 (3.39)		

Significant differences in bold

## 4 Discussion

In this study, a non-linear scaling technique has been combined with a procedure to reconstruct bones from incomplete or scattered geometry data for the first time. This method combines the advantages of non-linear scaling techniques, which have been successfully shown to predict muscle geometries based on bone shapes (Correa et al., 2011, Kaptein and van der Helm, 2004, Pellikaan et al., 2014) with the ability of statistical shape modelling to reconstruct bones to high accuracy. Reconstructions from point sets of partial bones reduced the accuracy of the predicted shape only marginally. This makes the presented method an alternative to linear landmark-based scaling and will be particularly useful in cases where medical images are obtained from partial bones, which is common practice for patients with knee osteoarthritis or patients considered for joint replacement.

The maximal RMSE of the bone reconstruction obtained here was approximately 0.5 mm greater than the longitudinal resolution of the medical images and therefore the reconstruction was assumed to be sufficiently accurate. Unfortunately, peak errors were observed close to bony landmarks; specifically, the errors around the malleoli were critical. This might have influenced the accuracy of the reconstruction of landmarks from a reference model and might be a reason why no significant difference between the reconstructions of the landmarks was observed. The calculated RMSEs between manually digitised and reconstructed surfaces to the mean shape showed that non-linear scaling has higher accuracy than the linear and the affine scaling methods. It also was shown that the proposed method was more accurate for the muscle geometry of the thigh. For the shank, the reduction of the errors was not significant. A reason for this can be found in the inter-subject variability of the muscle geometry which was also reported in Duda et al. (1996) using a linear scaling method and in Pellikaan et al. (2014) who used a non-linear scaling method and reported similar variability as obtained in this study. Possible extensions of the model could be to incorporate the variability into the reference models which would allow calculation with uncertainties in musculoskeletal simulations or the generation of SSMs of bones and muscle paths. For both methods, the number of subjects with digitised muscle paths in this study was too low to obtain powerful statistical models. This will be attempted in the future.

The increased accuracy in surface and muscle scaling of affine compared to linear scaling verifies its use as lower bound; the fact that errors were larger for landmarks might lie in the fact that the purpose of the linear scaling method was to match especially the landmarks. Due to the small differences between the non-linear scaling and manual digitisation methods, we expect that muscle force predictions using these geometries will also result in small differences.

In conclusion, it was shown that the accuracy of scaled muscle models using our non-linear scaling method is higher than linear scaled models.

## Conflict of interest statement

The authors do not have any financial or personal relationships with other people or organisation that could inappropriately influence their work.

## Footnotes

Appendix A Supplementary data associated with this article can be found in the online version at 10.1016/j.jbiomech.2016.09.005.
