# Supplementary material for: Non-linear scaling of a musculoskeletal model of the lower limb using statistical shape models
Source: J Biomech. 2016 Oct 3;49(14):3576–81. doi: 10.1016/j.jbiomech.2016.09.005 (PMC6399126; doi:10.1016/j.jbiomech.2016.09.005)
Supplement: Supplementary file 1 — Supplementary material [file mmc1.docx]

| **Subject** | **Femur** | **Tibia/Fibula** |
| --- | --- | --- |
| **MT2** | 0.0 | 4.0 |
| **MS** | 5.0 | -9.0 |
| **FS** | 10.0 | -10.0 |

**Table:** Manual corrections in z-direction (long axis) of the SSM coordinate system in mm for the registration of the points to the mean shape of the SSM. No manual corrections were applied for subjects not listed.
